# Improving the Referral Process Between Acute Wards and the Psychiatry Department at Tameside General Hospital

**DOI:** 10.1192/bjo.2022.275

**Published:** 2022-06-20

**Authors:** Claudia Bann, Sharon Yeung, Emmalene Fish

**Affiliations:** 1 Tameside and Glossop Integrated Care NHS Foundation Trust, Greater Manchester, United Kingdom; 2 Pennine Care NHS Foundation Trust, Greater Manchester, United Kingdom

## Abstract

**Aims:**

The project aims to address the barriers faced by the acute hospital and the psychiatry department in the referral process for a psychiatric opinion, at Tameside General Hospital (TGH). The Care Quality Commission (CQC) undertook a review of how people's mental health needs were met in acute hospitals in 2017 and concluded that there were barriers to this, for multifactorial reasons. Examples included: acute hospital staff not feeling adequately prepared to treat mental health conditions and lack of mental health care services 24/7. The current referral process at TGH for the acute hospital doctors requesting a psychiatric opinion presents a challenge for the referring doctor and psychiatry doctor in receipt of the referral. Many at the acute hospital have found the process of referral unclear, and many in the psychiatric department have found that referrals seldom contain sufficient information to determine whether a psychiatric review is required and whether it needs to be prioritised.

**Methods:**

To understand the specific difficulties encountered during the referral process, two questionnaires were created. One for TGH acute trust doctors and one for the psychiatry doctors, asking what the perceived barriers were and how these could be overcome. Data were collected between September and October 2021.

**Results:**

We obtained results from 17 acute trust doctors. The results revealed that most referring doctors found the referral process unclear. 100% agreed that they would benefit from guidance with the referral process e.g., a psychiatry specific referral form and/or a flow chart outlining the referral process. All responders wanted guidance around the roles and responsibilities of the psychiatric team in relation to the hospital setting.

We obtained results from 7 psychiatry doctors. Most were not satisfied with the referrals received. 100% would like to see a specific psychiatry referral form implemented in the acute hospital.

**Conclusion:**

Key findings were: the referral process is unclear, acute trust doctors don't feel well enough equipped to manage mental health concerns, referrals don't contain sufficient patient information, and that the acute trust doctors don't know where to ask for help. The project reflected earlier CQC findings.

After discussion with the acute trust, our action plan includes creating a psychiatry-specific referral form, to be distributed together with a flow chart which directs acute trust doctors to the appropriate source for psychiatric opinions. We also aim to join departmental and junior doctor teachings regularly to distribute and educate on the process.

